# Transovarial effects of pyriproxyfen on Japanese beetle (Coleoptera: Scarabaeidae) in turfgrass

**DOI:** 10.1093/jisesa/ieag035

**Published:** 2026-04-20

**Authors:** Shimat V Joseph, Sabrine Attia

**Affiliations:** Department of Entomology, University of Georgia, Griffin, GA, USA; Department of Entomology, University of Georgia, Griffin, GA, USA; Laboratory of Bioaggressors and Integrated Pest Management in Agriculture (LR14AGR02), National Agronomic Institute of Tunisia (INAT), University of Carthage, Tunisia

**Keywords:** insect growth regulator, *Popillia japonica*, reduced-risk, ornamental

## Abstract

The Japanese beetle, *Popillia japonica* Newman (Coleoptera: Scarabaeidae), is a serious pest of landscape trees, shrubs, and turfgrass in the eastern United States. Currently, adults and larvae of *P. japonica* are targeted using repeated insecticide applications. Pyriproxyfen, an insect growth regulator (IGR), whether applied topically to adult *P. japonica* or ingested by them, reduces egg viability. However, the transovarial effect of IGRs on adult *P. japonica* is inadequately studied. Thus, the objective of this study was to determine whether transovarial activity of pyriproxyfen affects F1 grubs (offspring) density. In 2023 and 2024, field studies were conducted after exposing adult *P. japonica* to the maximum label rate of pyriproxyfen. The treatments were (i) dipping in solution, (ii) feeding on treated foliage, (iii) combined dipping + feeding, and (iv) a nontreated control. Results showed that the number of grubs was significantly lower in the dipping + feeding treatment than in the nontreated control treatment in both years. These findings demonstrate the potential of pyriproxyfen as a transovarial management tool for *P. japonica* and provide insight for integrated pest management in turfgrass and ornamental landscapes.

## Introduction

Invasive insects exhibit high ecological plasticity, enabling them to establish, spread, and persist across a wide range of novel environments (Baldwin 1896). Consequently, many invasive insect species have become major threats to global agriculture, biodiversity, and ecosystem functioning, causing substantial economic losses and long-lasting ecological impacts (Bertelsmeier et al. 2025). Among these invasive insects, the Japanese beetle, *Popillia japonica* Newman (Coleoptera: Scarabaeidae), is a serious pest of landscape trees, shrubs, and turfgrass in the eastern United States (Althoff and Rice 2022). Native to the Japanese archipelago, it was first detected in New Jersey in 1916 (Dickerson and Weiss 1918). As of 2025, *P. japonica* is considered established in at least 10 countries across North America, Europe, and Asia, and in 28 U.S. states (Althoff and Rice 2022). This status applies to populations that have reached a self-sustaining level at which eradication is no longer the primary objective. Nevertheless, federal and state quarantines remain enforced in several infested states to regulate the interstate movement of soil, nursery stock, and other regulated articles, thereby preventing further spread into non-infested regions, particularly in the western United States. The Japanese beetle costs over $460 million annually in management costs, primarily for turfgrass, ornamental landscapes, and nursery production systems, including both larval and adult control measures (USDA 2015). Although this estimate remains widely cited, it may underestimate current expenditures, particularly in western states where ongoing containment and eradication efforts require substantial regulatory and treatment investments. Larval damage alone accounts for $234 million of this total, comprising $156 million in lost turf replacement and $78 million in control costs (USDA 2015). Adult *P. japonica* is highly polyphagous, feeding on over 700 plant species across numerous plant families (Bragard et al. 2018). Grubs feed extensively on the roots of grasses in lawns, golf courses, parks, and athletic fields (Shanovich et al. 2019). Larvae feed aggressively on the roots of grasses, pruning them off and reducing the plant’s ability to take up water. This results in turfgrass that becomes brown, wilted, and drought‑stressed, even when soil moisture is adequate (Potter and Held 2002; Althoff and Rice 2022).

In the United States, *P. japonica* completes one generation per year (Bragard et al. 2018; Joseph et al. 2025). Adults emerge from turfgrass in Georgia between mid-May and early June (Joseph et al. 2025). Females emerge a few days before males (Van Timmerman et al. 2001). The recently emerged females mate, then drop to the ground, burrow into the soil, and lay eggs (Potter and Held 2002). The oviposition bouts are repeated 12 times. They burrow into the soil and can deposit up to 60 individual eggs (Fleming 1972). Eggs hatch, and larvae develop in the soil, feeding on roots and organic matter (Althoff and Rice 2022). The larvae move deeper into the soil profile as they molt into higher instars during late fall and winter (Fleming 1976, USDA 2015, Gyeltshen et al. 2019, Joseph et al. 2025). They overwinter as larvae, in early spring (typically March to April in temperate regions of the United States), migrate to the upper 15 cm of the soil profile, feed again for 4 to 8 wk before pupating in late April to May, preceding adult emergence in May to June (Vittum 1986; Joseph et al. 2025).

Management of *P. japonica* larvae is challenging because they develop belowground and feed on plant roots, typically within the upper 5 to 15 cm of the soil profile during active feeding. However, larvae may migrate deeper, often reaching depths of 20 to 30 cm in response to declining soil temperatures, drought stress, or soil type, thereby reducing exposure to surface-applied control measures (USDA 2015, Vittum 1986).

A variety of insecticide classes are effective against *P. japonica*, including pyrethroids, organophosphates, carbamates, neonicotinoids, and anthranilic diamides (Larson et al. 2014, Piñero and Dudenhoeffer 2018). Pyrethroids, such as bifenthrin, cyfluthrin, deltamethrin, permethrin, and carbamates, such as carbaryl, are primarily recommended for adult management and provide rapid knockdown but often require repeated applications and may affect non-target organisms (USDA 2015). In contrast, neonicotinoids and diamides are widely used for larval suppression in turfgrass due to their systemic or soil activity; however, environmental concerns, particularly regarding pollinator exposure, have led to regulatory restrictions on certain neonicotinoids in ornamental systems in parts of the northeastern United States (Douglas and Tooker 2015). Because adults feed mainly on ornamental foliage, control efforts are typically directed at infested hosts, whereas larvae target turfgrass roots belowground.

Some insect growth regulators, such as halofenozide, have previously been commercialized for grub control in turfgrass and have demonstrated efficacy against *P. japonica* larvae (Mannion et al. 2000). However, IGRs are rarely used to manage adult and larval populations of *Popillia japonica*, primarily due to the limited availability of labeled products for grub control, their slower mode of action, and reduced efficacy against later larval instars compared with conventional insecticides. These insecticides primarily target immature stages and may play an important role in regulating *P. japonica* population development in landscape systems (Graf 1993). IGRs are considered reduced-risk compounds with low toxicity to non-target organisms, particularly mammals (Graf 1993). Among IGRs, pyriproxyfen is a pyridine-based juvenile hormone analog registered for ornamental plants and classified in Group 7C by the Insecticide Resistance Action Committee (IRAC 2025). Pyriproxyfen is widely used to control a range of insect pests, primarily fungus gnats, thrips, whiteflies, scales, and leaf miners in ornamentals (Smith et al. 2022). Although direct evidence for transovarial effects of pyriproxyfen in *P. japonica* remains limited, studies on other insect species have shown that adult female exposure to pyriproxyfen can affect the egg production or viability of insect eggs (Oouchi 2005).

Transovarial effects of IGRs are effective against many insect pests, such as the Colorado potato beetle, *Leptinotarsa decemlineata* (Say) (Coleoptera: Chrysomelidae) (Alyokhin et al. 2009), and the vine weevil, *Otiorhynchus sulcatus* (Fabricius) (Coleoptera: Curculionidae) (Zepp et al. 1979; Cowles 2004). However, the transovarial effects of IGRs, such as pyriproxyfen, have not been demonstrated in *P. japonica*. The objective of this study was to determine the transovarial effects of pyriproxyfen on adult *P. japonica*. If proven effective, this strategy can be used to spray adults feeding on ornamental shrubs to reduce egg-laying or egg viability among females landing on turfgrass, thereby reducing population size.

## Materials and Methods

### Study Site

In 2023 and 2024, experiments were conducted on ‘Tifway’ bermudagrass at the University of Georgia, Griffin, Georgia. The bermudagrass was mowed weekly at a height of 8 cm and irrigated daily for 30 min. The experimental area was fertilized, and herbicide (Monosodium Methanearsonate, Drexel Chemical Company, and Luxembourg-Pamol, Inc., Memphis, Tennessee) was applied at 0.32 ml per m^2^ as a post-emergent organic arsenical herbicide to control grassy and broadleaf weeds, such as crabgrass [*Digitaria sanguinalis* (L.) Scop], and white clover (*Trifolium repens* L.) a month before initiation of the experiment in both years. However, no insecticide was applied. Still, over time, 10% of the bermudagrass field became infested with grassy weeds, and treatments were applied only where bermudagrass remained continuously present.

### Insect

Japanese beetles were field-collected from a corn-soybean farm (Bledsoe Research Farm, UGA Griffin Campus) using commercial lure with eugenol (21.98%), Geraniol (9.43%), 2-Phenyl Ethyl Propionate (9.43%), (R, Z)-5-(1-Decenyl) dihydro-2(3H) furanone (0.02%) and other ingredients (59.14%) (Spectracide Bag-A-Bug Japanese Beetle Trap_2_, Combo lure, Spectrum Group, United Industries Corporation, St. Louis, Missouri). The collected live beetles were maintained for 2 to 3 d in 4 clear plastic boxes (30 × 46 × 17.8 cm; Sterilite, Townsend, Massachusetts) on crape myrtle foliage with proper aeration. Freshly collected crape myrtle foliage was provided on the third day. During this period, individuals were classified as male or female. Males possess a shorter, more robust tarsus, whereas females exhibit a longer tarsus accompanied by a more rounded tibial spur (Kelly 2020).

### Insecticide

Pyriproxyfen (Fulcrum [11.2% a.i.], OHP Inc., Bluffton, South Carolina, United States) at 90.3 g per ha was used in the study. A water volume of 373.9 l/ha was used to prepare a pyripro­xyfen solution at 241.7 ppm. Adjuvants or surfactants were not used.

### Experiment Design and Evaluation

The treatments were exposure to pyriproxyfen, which included (i) dipping, (ii) feeding, (iii) dipping + feeding, and (iv) nontreated control. Adult females were dipped in the pyriproxyfen solution for 6 s in the dipping treatment. For the feeding treatment, adults were allowed to feed for 24 h on crape myrtle foliage that had been sprayed with a pyriproxyfen solution using a pneumatic sprayer and plant material air-dried for 24 h. For the dipping + feeding treatment, adults were first allowed to feed on pyriproxyfen-treated foliage for 24 h, then dipped in a pyriproxyfen solution for 6 s. For pyriproxyfen exposure, 20 females and 10 males were sorted into 20 ml sample cups. After exposure, the beetles were reintroduced to the same cup with a non-treated crape myrtle leaf as food. The lid of the sample cup had 4 4-mm-diameter holes for ventilation. The treated beetles were introduced to each tube in the field.

**Fig. 1.**
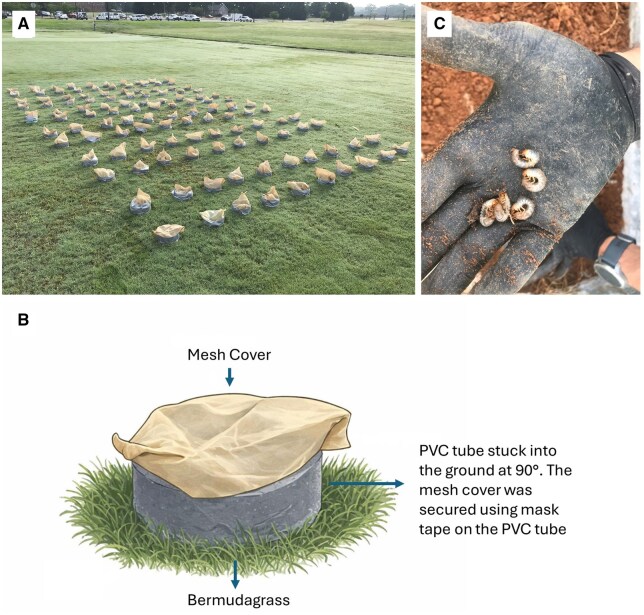
A) The experimental setup of the *Popillia japonica* grub experiment in the turfgrass field, B) a diagram of a tube positioned in the ground, and C) grubs collected from the tubes.

The experiments were conducted on 2 different areas of the ‘Tifway’ bermudagrass field in both years. Six replicates of each treatment were assigned according to a randomized complete block design. The plot size was 0.3048 m × 0.6096 m. Two 30 cm long × 0.02 m^2^ diameter PVC tubes were deployed on the ground in each plot using a sledgehammer and a wooden bolt. Approximately 20 cm of the tube was buried in the soil. On 8 June 2023 and 12 June 2024, each tube received 30 pre-pyriproxyfen-exposed adult beetles (20 females + 10 males), and thus, each replicate received 60 beetles. These 2 tubes served as an experimental unit. The introduction procedure was performed by emptying beetles from each cup into the corresponding tube and securing the screen mesh to the PVC tube with a strip of masking tape around the lip to prevent the beetles from flying (Fig. 1A). Beetles were allowed to mate and oviposit for 7 d within the tubes. After 7 d, the screen-mesh from each tube was removed. The plots were maintained with regular irrigation throughout the experimental area as described in the study site section. The grass growing in and around the tubes was mowed with a weed eater at 4-wk intervals. On 6 September 2023 and 9 October 2024, the tubes were retrieved from the ground, and the live second- and third-instar individuals (Fig. 1B) were carefully extracted from each tube and quantified.

### Statistical Analysis

All data from the 2 experiments were analyzed in SAS (SAS Institute 2024). To determine the effect of pyriproxyfen on live grubs, the *P. japonica* grub data were natural log-transformed (ln[*x* + 1]) and analyzed using a general linear model (PROC GLM) with pyriproxyfen treatment, year, their interaction, and replication as factors. Because the year was a significantly different factor, analysis was performed by year to determine the effect of pyriproxyfen on live grubs. The normality of the data was checked by assessing residuals using PROC UNIVARIATE in SAS. The means were separated using Tukey’s HSD test for treatment comparisons. All the statistical comparisons were considered significant at α = 0.05.

## Results and Discussion

The factors year and pyriproxyfen treatment were significantly different for live *P. japonica* grubs collected from the tubes (Table 1). However, the year × treatment interaction and replication were not significantly different (Table 1). In 2023, the number of *P. japonica* grubs was significantly lower for the dipping + feeding treatment than for the feeding and nontreated control treatments (*F *= 5.0; df = 3, 15; *P *= 0.013; Fig. 2A). There was no significant difference in the number of *P. japonica* grubs between the dipping and dipping + feeding treatments. In 2024, significantly fewer *P. japonica* grubs were observed in the dipping + feeding treatment than in the nontreated control (*F *= 4.8; df = 3, 15; *P *= 0.016; Fig. 2B).

**Figure:**
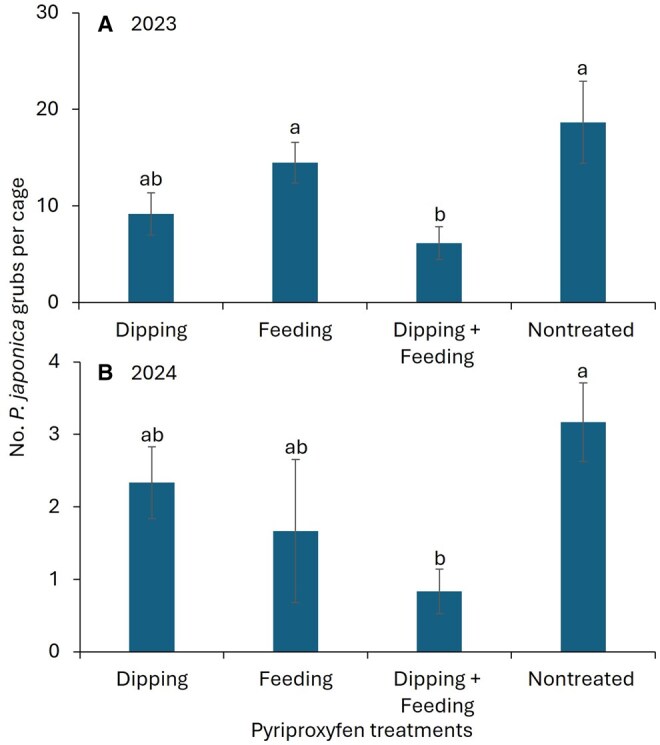
Fig. 2. Mean (±SE) number of live *Popillia japonica* grubs recovered from the cage after adults were exposed to pyriproxyfen by various methods in A) 2023, and B) 2024. The same letters on the bars indicate no significant difference among treatments using Tukey’s HSD test (*P *< 0.05).

**Table:** Table 1. Statistical analysis to determine the effects of treatments on *Popillia japonica* grub recovered from the tube

Variable	*F*	df	*P*
**Year**	25.4	1, 35	<0.001
**Treatment**	6.5	3, 35	0.001
**Year × treatment**	1.8	3, 35	0.157
**Replication**	1.5	5, 35	0.217

The results suggest that pyriproxyfen can reduce *P. japonica* grub densities when adults are exposed to it. Previously, when the transovarial effects of pyriproxyfen were examined after topical spray on adult tingids, *Stephanitis pyrioides* Scott and *Teleonemia scrupulosa* Stål, a reduced number of nymphs were observed (Joseph 2019, 2022).

Pyriproxyfen applied to adult *P. japonica* and allowed to feed on treated leaves can reduce egg production or hatch; however, the dipping + feeding treatment may expose beetles to levels higher than those in typical field sprays, so field effects are expected to be lower. Neither topical exposure nor feeding alone significantly affected egg production or viability. Adult *P. japonica* must feed on and come into contact with pyriproxyfen residues to achieve an effective reduction in F1 grubs. These findings indicate that effective reproductive ­disruption in *P. japonica* requires combined contact and ingestion of pyriproxyfen, likely through interference with juvenile hormone-regulated processes during oogenesis and embryonic development (Dhadialla et al. 1998). Targeting adults to suppress larval populations indirectly is particularly relevant, as grubs cause the most severe damage to turfgrass root systems (Potter and Held 2002). Within an integrated pest management framework, pyriproxyfen is a selective option that may reduce reliance on soil-applied insecticides (Joseph 2022).

Further research is warranted to determine whether the reduction in egg production and hatch resulting from pyriproxyfen exposure of adult *P. japonica* will ultimately reduce grub populations and lower the risk of turfgrass being damaged, as fewer grubs feed on turfgrass roots. Over the years, repeated applications of pyriproxyfen can suppress the *P. japonica* population, thereby reducing adult damage to shrubs. However, repeated use of pyriproxyfen or other IGRs could select for resistant populations, emphasizing the importance of rotating modes of action within integrated pest management programs. Further research is needed to determine whether other IGRs can produce comparable or superior results. It should be noted, however, that our study likely exposes adults to higher levels of IGR than would occur under field conditions, where beetles can avoid sprays and feed on a mixture of treated and untreated foliage, which may reduce the effectiveness of these compounds. Additionally, the use of IGRs on flowering ornamentals such as crape myrtle may pose risks to non-target insects, including pollinators and natural enemies, as juvenile hormone analogs can affect reproduction and development in a range of insect species. The activity of female *P. japonica* declines by late May in certain regions of the southeastern states, while some crape myrtle cultivars begin flowering, reaching a peak of flowering in July. IGRs may be integrated into existing management programs to enhance pest suppression and reduce overall insecticide use, thereby lowering risks to humans, non-target organisms, and the environment without affecting the quality of turfgrass or ornamental shrubs.

## References

[ieag035-B1] Alyokhin AR , Guillemette R, Choban R. 2009. Stimulatory and suppressive effects of novaluron on the Colorado potato beetle reproduction. J. Econ. Entomol. 102:2078–2083. 10.1603/029.102.0609 20069834

[ieag035-B2] Althoff ER , Rice KB. 2022. Japanese beetle (Coleoptera: Scarabaeidae) invasion of North America: history, ecology, and management. J. Integr. Pest Manage. 13:2. 10.1093/jipm/pmab043

[ieag035-B3] Baldwin JM. 1896. A new factor in evolution. Am. Natural. 30:441–451. 10.1086/276408

[ieag035-B4] Bertelsmeier C , Bonnamour A, Garnas JR, et al 2025. Temporal dynamics and global flows of insect invasions in an era of globalization. Nat. Rev. Biodivers. 1:90–103.

[ieag035-B5] Bragard C , Dehnen-Schmutz K, Di Serio F, et al 2018. Pest categorisation of *Popillia japonica*. EFSA Panel on Plant Health (PLH). Scientific Opinion. 10.2903/j.efsa.2018.5438

[ieag035-B6] Cowles RS. 2004. Impact of azadirachtin on vine weevil (Coleoptera: Curculionidae) reproduction. Agric. Forest Entomol. 6:291–294. 10.1111/j.1461-9555.2004.00235.x

[ieag035-B7] Dhadialla TS , Carlson GR, Le DP. 1998. New insecticides with ecdysteroidal and juvenile hormone activity. Annu. Rev. Entomol. 43:545–569. 10.1146/annurev.ento.43.1.545 9444757

[ieag035-B8] Dickerson EL , Weiss HB. 1918. *Popilia japonica* Newm., a recently introduced Japanese pest. Can. Entomol. 50:217–221.

[ieag035-B9] Douglas MR , Tooker JF. 2015. Large-scale deployment of seed treatments has driven rapid increase in use of neonicotinoid insecticides and preemptive pest management in U.S. field crops. Environ. Sci. Technol. 49:5088–5097. 25793443 10.1021/es506141g

[ieag035-B10] Graf JF. 1993. The role of insect growth regulators in arthropod control. Parasitol. Today 9:471–474. 15463697 10.1016/0169-4758(93)90106-p

[ieag035-B11] Gyeltshen J , Hodges A, Bania C. 2019. Japanese beetle, Popillia japonica Newman (Insecta: Coleoptera: Scarabaeidae) (EENY350). University of Florida, IFAS Extension. [accessed 2025 Dec 28]. https://edis.ifas.ufl.edu/publication/IN630/pdf

[ieag035-B12] Fleming WE. 1972. Biology of the Japanese beetle. Technical Bulletin No. 1449. Washington, DC: USDA.

[ieag035-B13] [IRAC] Insecticide Resistance Action Committee. 2025. [accessed 2025 Dec 28]. http://www.irac-online.org/modes-of-action/

[ieag035-B14] Larson JL , Redmond CT, Potter DA. 2014. Impacts of a neonicotinoid, neonicotinoid-pyrethroid premix, and anthranilic diamide insecticide on four species of turf-inhabiting beneficial insects. Ecotoxicology 23:252–259. 10.1007/s10646-013-1168-4 24493235

[ieag035-B15] Ishaaya I , Horowitz AR. 1995. Pyriproxyfen, a novel insect growth regulator for controlling whiteflies: mechanisms and resistance management. Pestic. Sci. 43:227–232. 10.1002/ps.2780430308

[ieag035-B16] Joseph SV. 2017. Effects of insect growth regulators on *Bagrada hilaris* (Hemiptera: Pentatomidae). J. Econ. Entomol. 110:2471–2477. 10.1093/jee/tox264 29040567

[ieag035-B17] Joseph SV , Hudson WG, Braman SK, et al 2025. Japanese beetles in the nursery and landscape. University of Georgia Cooperative Extension Circular 1167. [accessed 2025 Dec 28]. https://fieldreport.caes.uga.edu/wp-content/uploads/2025/08/C-1167_3.pdf

[ieag035-B18] Joseph SV. 2019. Transovarial effects of insect growth regulator on *Stephanitis pyrioides* (Hemiptera: Tingidae). Pest Manag. Sci. 75:2182–2187. 10.1002/ps.5342 30653837

[ieag035-B19] Joseph SV. 2022. Insect growth regulators elicit transovarial effects to *Teleonemia scrupulosa* (Hemiptera: Tingidae). Pest Manag. Sci. 78:1800–1805. 10.1002/ps.6797 35019229

[ieag035-B20] Kelly CD. 2020. Sexual selection on size and shape in Japanese beetles (*Popillia japonica*). Behav. Ecol. 31:1073–1083. 10.1093/beheco/araa054

[ieag035-B21] Mannion CM , Winkler HE, Shapiro DE, et al 2000. Interaction between halofenozide and the entomopathogenic nematode *Heterorhabditis marelatus* for control of Japanese beetle (Coleoptera: Scarabaeidae) larvae. Biol. Microb. Control 39:48–53. 0022-0493/00/0048Ð0053$02.00/0 10.1603/0022-0493-93.1.48 14658511

[ieag035-B22] Oouchi H. 2005. Insecticidal properties of a juvenoid, pyriproxyfen, on all life stages of *Plutella xylostella* (Lepidoptera: Yponomeutidae). Appl. Entomol. Zool. 40:145–149. 10.1303/aez.2005.145

[ieag035-B23] Piñero JC , Dudenhoeffer AP. 2018. Mass trapping designs for organic control of the Japanese beetle, *Popillia japonica* (Coleoptera: Scarabaeidae). Pest Manag. Sci. 74:1687–1693. 10.1002/ps.4862 29337404

[ieag035-B24] Potter DA , Held DW. 2002. Biology and management of the Japanese beetle. Annu. Rev. Entomol. 47:175–205. 10.1146/annurev.ento.47.091201.145153 11729073

[ieag035-B25] Pyriproxyfen General Fact Sheet. National Pesticide Information Center, Oregon State University Extension Services. [accessed 2025 Dec 28]. http://npic.orst.edu/factsheets/pyriprogen.html

[ieag035-B26] SAS Institute. 2024. SAS Version 9.4. Cary, NC: SAS Institute Inc.

[ieag035-B27] Shanovich HN , Dean AN, Koch RL, et al 2019. Biology and management of Japanese beetle (Coleoptera: Scarabaeidae) in corn and soybean. J. Integr. Pest Manage 10:9. 10.1093/jipm/pmz015

[ieag035-B28] Smith H , Dale A, Beuzelin J. 2022. Understanding insecticide modes of action and resistance management in Florida Horticulture. IFAS Extension.P8 [accessed 2026 Jan 2]. 10.32473/edis-IN1379-2022

[ieag035-B29] [USDA] U.S. Department of Agriculture, Animal and Plant Health Inspection Service. 2015. Managing the Japanese beetle: a homeowner’s handbook. [accessed 2025 Dec 8]. https://www.govinfo.gov/app/details/GOVPUB-A-PURL-gpo80858

[ieag035-B30] Van Timmerman SJ , Switzer PV, Kruse KC. 2001. Emergence and reproductive patterns in the Japanese beetle, *Popillia japonica* (Coleoptera: Scarabaeidae). J. Kansas Entomol. Soc. 74:17–27. https://www.jstor.org/stable/25085984

[ieag035-B31] Vittum PJ. 1986. Biology of the Japanese beetle (Coleoptera: Scarabaeidae) in eastern Massachusetts. J. Econ. Entomol. 79:387–391. 10.1093/jee/79.2.387

[ieag035-B32] Zepp DB , Dierks AZ, Sanders DJ. 1979. Effects of diflubenzuron on black vine weevil oviposition, egg viability, and adult longevity (Coleoptera: Curculionidae). J. Kansas Entomol. Soc. 52:662–666. https://www.jstor.org/stable/25083979

